# Pain and Stress Response during Intravenous Access in Children with Congenital Adrenal Hyperplasia: Effects of EMLA and Nitrous Oxide Treatment

**DOI:** 10.1155/2017/1793241

**Published:** 2017-12-31

**Authors:** K. Ekbom

**Affiliations:** ^1^Division of Paediatrics, Department for Clinical Science Intervention and Technology, Karolinska Institute, Karolinska University Hospital, 141 86 Stockholm, Sweden; ^2^Division of Endocrinology, Diabetes and Metabolism, National Childhood Obesity Centre, Karolinska University Hospital, 141 86 Stockholm, Sweden

## Abstract

**Background:**

Congenital adrenal hyperplasia (CAH) is an endocrine condition that requires regularly blood samples for optimal treatment. The management of CAH in children is complex when intravenous access is one of the most stressful procedures for children. The purpose of this pilot study was to investigate the effects of nitrous oxide inhalation (N2O) in combination with cutaneous application of local anesthetics (EMLA) for improving intravenous access in children with CAH.

**Method:**

Ten children (7–14 years) were studied. The children received two intravenous procedures: one with EMLA and one with EMLA + N2O. The order of priority was randomized. The outcomes were the children's pain experience (0–10) and an evaluation of satisfaction (1–5) after the procedure. Heart rate, blood pressure, saturation, and analyses of 17-hydroxyprogesterone (17-OHP), norepinephrine, and glucose were analyzed.

**Results:**

Higher pain scores, heart rate, and glucose levels were reported after EMLA, compared to EMLA + N2O, but 17-OHP levels remained unchanged. The children's satisfaction with the intravenous procedure was more positive for EMLA + N2O.

**Conclusions:**

EMLA + N2O offers the possibility of improving the intravenous procedure for anxious children with CAH. Although the quality of care was better with N2O treatment, it was not possible to demonstrate that this is a prerequisite for valid 17-OHP measurements.

## 1. Introduction

Congenital adrenal hyperplasia (CAH) is one of the most prevalent monogenetic autosomal recessive endocrine disorders [1, 2]. Ninety-five percent of all cases are caused by a deficiency in 21-hydroxylase, which results in impaired biosynthesis of cortisol and cortisol deficiency as well as androgen excess with or without aldosterone deficiency. CAH shows a range of severity and is clinically classified into classic forms, the severe form, divided into simple virilizing and salt-wasting forms (33% and 67%, resp.), or nonclassic forms, the mild or late-onset form. Data from newborn infants, screened in 13 countries including Sweden, show an incidence of one in 15,000 livebirths for the classic form of CAH and an incident about one in 1,000 for the nonclassic form [3–5].

Medical treatment for CAH using glucocorticoids needs to be individualized to suppress androgen secretion, yet without total suppression of the hypothalamic-pituitary-adrenal (HPA) axis, which requires high glucocorticoid doses that cause negative side effects [6–10].

The 17-hydroxyprogesterone (17-OHP) levels are used to adjust the hydrocortisone dose and stressful IV access may affect 17-OHP levels, which could be interpreted as a sign of an insufficient glucocorticoid substitution dose.

The management of CAH is complex and adequate monitoring of therapy is important and includes IV access. However, there are other valuable control parameters, such as 17-OHP in saliva or urinary steroids (pregnanetriol), compared to the measurement of serum 17-OHP alone. However, an adequate monitoring includes as well blood samples for analysis of serum androstenedione, indicating if the children have appropriately therapy.

The target of 17-OHP and other hormones vary widely in children depending on the time of day and the interval since the last glucocorticoid dose. A concentration of 17-OHP >40 nmol/L indicates undertreatment [11]. Overtreatment can lead to growth suppression and potentially obesity as well as osteoporosis [7, 8, 10]. Since undertreatment results in an excess of high androgens, close control of medication is necessary [7, 12].

The use of intravenous (IV) access procedures is common in all chronically ill children [13], and IV procedures have been ranked as some of the most unpleasant procedures that children must undergo at a hospital [14]. Stress factors in connection with IV access include both emotional/physical stress and often poor vein selection [15].

Pain relief before venipuncture is an important issue for all anxious children and/or children with poor quality veins. We previously compared the efficiency of the standard procedure using EMLA with EMLA+ inhaled 50% nitrous oxide (N2O) in children with difficulties related to IV access [16]. However, we do not know how anxious children with CAH evaluate the IV procedure using conventional pain relief with EMLA and whether treatment with N2O would be a suitable alternative treatment for these children. Inhalation of nitrous oxide (N2O) is a well-established method and has both pain reduction and sedation effects [17, 18], which may be useful when treating anxious children with CAH.

It is even unclear if procedural pain in connection with IV access affects the analysis of 17-OHP and that connection has not been studied before in children with CAH.

The aim of our study was to compare EMLA with EMLA + N2O in children with CAH reporting anxiety in establishing IV access. We hypothesized that EMLA + N2O would be superior to EMLA reducing pain in children with CAH undergoing IV access and that the pain and anxiety reductions effects of N2O would decrease children's physiological stress markers and adrenal hormone secretion. Children's evaluation of pain, satisfaction with the IV procedure, and children's physiological stress markers were the primary end point. Secondary end point was levels of 17-OHP, norepinephrine, glucose, parents, and nurses satisfaction with the IV procedure.

The study was approved by the Ethical Committee of South Stockholm (Decision 050114).

*Trial Registration*. Current controlled trial is registered with ISRCTN33779750 (controlled-trials.com) and Karolinska Clinical Trial Registration (KCTR) is registered with CT 20090023.

## 2. Method

### 2.1. Design

**Figure 1:**
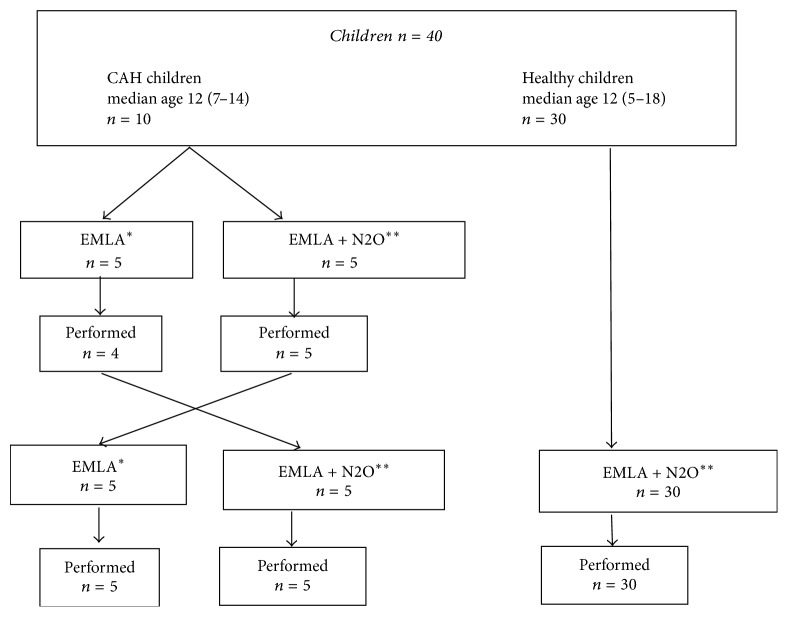
Flow diagram of the included children randomly allocated to two procedures, EMLA, and EMLA + N2O. ^*∗*^Inhalation of 100% oxygen. ^*∗∗*^Inhalation of 50% nitrous oxide.

The study design was a randomized, double-blind, crossover study including 10 children with CAH. The children were randomly allocated to two procedures. One procedure with EMLA and the inhalation of 100% oxygen (EMLA) and one procedure with EMLA and the inhalation of 50% nitrous oxide (EMLA + N2O), Figure 1.

One criterion for inclusion was that children had experienced previous difficulties connected to establishing IV access. The difficulties were defined as either a previous requirement of several attempts before establishing IV access or children rating anticipatory anxiety > 4 using a numeric rating scale (0–10) with 0 = no anxiety and 10 = worst possible anxiety [19, 20].

To be included, children also needed to have the ability to understand and comply with the different treatments, including using a face mask and interpreting the numeric rating scale (0–10) and Likert Scale (1–5) with verbal categorical response options of satisfaction with the IV procedure.

In addition to the double-blind crossover study, 30 children aged 5–18 served as controls. These children underwent blood sampling at the same time points after nitrous oxide sedation.

A research nurse randomized the children and prepared the anesthesia machine. She also set the percentage of nitrous oxide and oxygen according to randomization and concealed the mixing device. A pediatric nurse performed all the IV procedures and when the child had started to breathe into the mask for three minutes, the nurse set up an IV line.

The children, parents, and nurses were unaware of the randomization codes until data entry was completed. The procedure has been described in detail elsewhere [21].

#### 2.1.1. Anesthesia Equipment

Oxygen and nitrous oxide were mixed using the Engstrom 2024® (Engstrom Medical AB Stockholm, Sweden) connected to nitrous oxide and oxygen sources.

#### 2.1.2. Procedure

The children received EMLA® cream (Lidokain Prilokain 25 mg/g Astra Zeneca Södertälje, Sweden) 1 hour before IV access. They were also informed that during one of the procedures N2O treatment would be administered. All children were breathing a gas mixture continuously administered by an anesthesia machine through a mask from 3 minutes before until 3 minutes after the procedure. The anesthesia machine was hidden from the children and investigators by the research nurse. The IV cannulation was performed by an experienced pediatric nurse using a 22 G catheter.

The last dose of hydrocortisone was given in the morning, and no medication doses were adjusted between the 2 IV procedures.

### 2.2. Data Collection

All procedures were performed and blood samples were collected between the hours of 8:00 and 10:00. Blood samples were taken by venipuncture 5 minutes after venous access. Samples taken for plasma (P-) glucose (mmol/L), serum (S) androstenedione (nmol/L), and P-norepinephrine (NE; nmol/L) were analyzed directly in the Karolinska University Hospital laboratory. Samples obtained for S-17-OHP (nmol/L) were centrifuged immediately and stored at –80°C until they were assayed.

S-Androstenedione was analyzed, to measure if children had appropriately therapy.

30 children aged 5–18 served as controls, treated with EMLA + N2O.

Fifteen minutes after the IV procedure, the children were asked to rate the pain experience during IV access by using a numeric rating scale (NRS) of 0–10 with zero as no pain and 10 as the worst possible pain [20]. The children, parents, and nurses were also asked to do an evaluation, that is, the satisfaction with the procedure itself on a 5-point Likert Scale (1–5) in which 1 was poor, 2 was fair, 3 was good, 4 was very good, and 5 was excellent [22]. The children performed their evaluations first, and then the parents were asked to evaluate the procedure. The parents were present when the children made their assessments.

Additional variables recorded by the investigator included age, body mass index standard deviation score (BMI SDS) [23], the number of attempts, heart rate, blood pressure (NAIS-Blood Pressure Watch Diagnostic®), and oxygen saturation using pulse oximetry (Date-Ohmeda TUFF SAT®). Hypoxia was defined as a saturation of less than 93%. All side effects were documented in the study protocol.

### 2.3. Analysis

P-Glucose levels were measured using Modular Analytics P (Roche Diagnostics GmbH, Mannerheim, Germany). S-Androstenedione levels were measured using chromatography and mass spectrometry. P-NE was determined using HPLC (High Pressure Liquid Chromatography) (Dionex Corporation, Sunnyvale, CA, USA). The 17-OHP in serum was measured using a commercially available enzyme immunoassay (ELISA kit, Labor Diagnostic Nord Gmb & Co., KG, Nordhorn, Germany).

#### 2.3.1. Statistical Analysis

All statistical analyses were performed using SPSS for windows version 22 (SPSS, IL, USA).

Descriptive data are presented as median values and ranges. The treatment groups were compared using nonparametric statistics. For treatment comparisons of pain, evaluations, heart rate, blood pressure, saturation, and median values of blood samples of paired data the Wilcoxon test was used. When comparing CAH children with the control group independent *t*-test was used. Correlations were assessed using Spearman's rank order correlation.

The body mass index standard deviation score was calculated according to the study by Rolland-Cachera et al. [23]. All tests were two-sided, and *p* values <0.05 were regarded as statistically significant.

## 3. Results

The demographic characteristics and blood analyzes of the children that were studied are summarized in Table 1.

Three children were classified as nonclassic (NC) CAH, and 7 were classified as classic severe form (2 simple virilizing form and 5 salt-wasting form) [3] and the hydrocortisone doses varied from 7.8 to 17.0 mg/m^2^/day, data not shown.

The hydrocortisone doses varied from 7.8 to 17.0 mg/m^2^/day. Three children were classified as nonclassic CAH, mean dose of 10,9 mg/m^2^/day, and 7 were classified as classic severe form, mean dose 12,2 mg/m^2^/day (2 simple virilizing forms and 5 salt-wasting forms, mean dose 13,7 and 11,6 mg/m^2^/day, resp.).

A higher pain score was reported after EMLA compared to EMLA + N2O (*p* = 0.02), and the children's evaluations of satisfaction with the IV procedure were more positive for the EMLA + N2O treatment compared to EMLA (*p* = 0.01). Parents and nurses considered treatment with EMLA + N2O to be significantly better.

The heart rate increased significantly during IV access (*p* = 0.02) when using EMLA compared with EMLA + N2O as well as a trend of higher blood pressure. No differences were seen in saturation levels before, during, or after the IV access when comparing the different treatments. We found a significant correlation between children's evaluation of pain and number of attempts for a successful IV access (*r* = 0.6) data not shown.

The level of androstenedione was 1.3 (0.6–11.0), which indicated the children were receiving an appropriate dose.

No differences in levels of 17-OHP and NE after IV access were seen after comparing EMLA and EMLA + N2O, with no differences between children with salt-wasting and simple virilizing forms. However, glucose levels were significantly lower after the EMLA + N2O treatment (*p* = 0.04), as shown in Table 1.

No correlation was found between levels of 17-OHP and children's evaluation of pain.

Levels of 17-OHP were also measured in a control group of 30 healthy children who received IV access through EMLA + N2O. Significant lower glucose levels were seen in CAH children (*p* < 0.01), with no difference in the reported pain score between CAH children and control children.

In the EMLA group, IV access was not achieved in one patient, and the procedure was stopped by the child after the first blood sample. IV access was achieved in all procedures when EMLA + N2O was used. No respiratory adverse events were seen and no other side effects were reported.

## 4. Discussion

The management of CAH is complex and adequate monitoring of therapy must include IV access. A clinical problem often arises for children, parents, and nurses when IV access has to be done in anxious CAH children who have poor quality veins. The IV procedure has even led discussions about whether the procedural stress affects the blood analysis and subsequent treatment.

In this randomized, double-blind crossover study, self-reported pain and stress markers as well as heart rate, blood pressure, and oxygen saturation were studied after two different procedures associated with IV access were conducted in distressed children with CAH.

Higher self-reported pain and lower satisfaction with the procedures were found after EMLA compared to EMLA + N2O, which was similar to the results of our previous studies [16, 21].

We also measured the evaluations of the IV procedure made by parents and nurses because nurses often take an active role and assist CAH patients and their parents with management of the condition [24]. Overall, the results from children, parents, and nurses showed clearly that they considered the procedure with EMLA to be difficult, which indicated there is a need for a better method for the IV procedure. The studied children's evaluation of pain and satisfaction of IV procedure were similar to results we obtained from evaluations made by obese children on the difficulties of the IV procedure [21].

To involve children, parents, and nurses before and during stressful procedures is necessary, and requests from the family as well as caregivers for good quality of care must be taken into account [25]. Our results clearly showed a possible method for improving IV procedures in anxious children with CAH. Treatment with N2O is easy to perform, and the equipment required includes an anesthetic block, a suction unit, a scavenging system, and a pulse oximeter. Nitrous oxide administration can easily be performed by a single, specially trained nurse if local regulations allow it.

Heart rate and glucose levels were higher when using EMLA, which probably occurred due to a higher stress response. However, number of attempts may be a confounding factor when measuring stress response in anxious children, but the low number of participants in our study does not allow correction for any confounding variables.

No differences in 17-OHP or NE levels were found when comparing the different treatments, which may indicate that clinically relevant 17-OHP levels can be measured despite higher stress markers and pain in connection with IV access. Thus, the values obtained after EMLA varied more than after EMLA + N2O, and it is therefore possible that, for some patients, optimal 17-OHP measurements require good pain relief. The control group of healthy children treated with EMLA + N2O had significantly higher glucose levels compared to CAH children treated with EMLA + N2O. This result coincided with results from a controlled trial that compared CAH adolescents with healthy volunteers to examine their response to high-intensity exercise. The report's authors concluded that patients with classic CAH have an impaired stress-induced capacity, which led to a defective glucose response [26].

Treatment with N2O has a well-known sympathetic effect [27], but no differences in NE levels between EMLA and N2O + EMLA were seen in the children in our study. One explanation for this lack of differences may be that patients with CAH have a reduced capacity to upregulate their sympathetic nervous system [26]. It is also possible that the pain-induced stress that occurred when EMLA was used increased NE secretion to the same levels as seen after N2O inhalation.

A placebo effect may have initiated when the children were informed that during one of the procedures they should be given a treatment which may decrease the pain of venipuncture. We know the placebo effect in children to be high [28], but we considered it unethical not to inform the child about the expected effect of N2O. However, the effects of expectations in children and adolescents, as well as the mediation by parents, need further investigation. Even specific clinical trial-designs for children have been suggested [29].

### 4.1. Strengths and Limitations of the Study

The principle strength of our study was its randomized, double-blind crossover design.

A weakness was the low number of patients included in the study. The small number was due to, first, CAH being a rare disease and, second, to the inclusion of only anxious children who experienced previous difficulties with IV access.

The children were asked to evaluate the procedure in front of their parents and it is possible that children's evaluation affects the parental evaluation. It is also well-known that parents' values and attitudes influence their children's pain evaluation [30]. Thus, when the study was designed it was considered impossible to separate parents and children after the IV procedure. All evaluations were performed in the same order throughout the study.

The BMI SDS was higher in the control group. However, the pharmacological distribution of N2O is marginally affected of body weight when N2O has a low solubility in blood, leading to rapidly increase of alveolar concentration and a rapidly decrease after the inhalation [31].

We can only speculate how the stress response may have been without any pain relief and whether any difference in 17-OHP levels could have been detected.

As mentioned before, optimizing the hydrocortisone dosage has major clinical importance [3]. Excess glucocorticoids suppress growth [7, 10], and the use of hydrocortisone has also been shown to have a positive correlation with BMI SDS. In other words, children with CAH have a higher risk of obesity [32]. On the other hand, suboptimal glucocorticoid doses may lead to reduced physical and mental capacity [33] as well as a high concentration of sex steroids, which may induce premature epiphyseal closure for the child [10].

Continued care, including frequent blood sampling, is required for children with CAH to prevent long-term adverse consequences from the disease, including metabolic syndrome and osteoporosis [7]. Treatment with N2O offers the possibility of improving the IV access procedure for children with CAH.

## 5. Conclusion

The management of CAH is complex and adequate monitoring of therapy is important.

Despite higher self-reported pain, heart rate, glucose levels, and lower satisfaction with the IV procedure after EMLA compared to EMLA + N2O, no difference in 17-OHP levels was found in children with CAH. Thus, although the quality of care was better with N2O treatment, it was not possible to demonstrate that this is a prerequisite for valid 17-OHP measurements.

## Figures and Tables

**(a):** 

	EMLA	EMLA + N2O	*p* ^*∗*^
*CAH children*	(*n* = 10)		
*Age*, year	12 (7–14)		
*Gender*, male/female	3/7		
*BMI SDS*	0.38 (0–6.8)		

*Attempts*, number	1.0 (1–5)	1.0 (1-1)	0.3
*Pain*, 0–10	4.5 (2–10)	1.5 (1–3)	*0.02*
*Children's* evaluations 1–5	3.0 (1–5)	5.0 (4-5)	*0.01*
*Parent's* evaluations, 1–5	3.0 (1–5)	5.0 (4-5)	*0.02*
*Nurse's* evaluations, 1–5	2.5 (1–5)	5.0 (4-5)	*0.02*
*Blood analyses*			
*17-OHP* nmol/L	5.9 (1.7–52.3)	5.3 (2.9–24.2)	0.9
*Androstenedione* nmol/L	1.9 (0.3–12.0)	1.3 (0.6–11.0)	0.7
*Glucose* mmol/L	4.5 (4.1–5.6)	4.1 (3.7–5.0)	*0.04*
*Norepinephrine* nmol/L	1.3 (0.5–2.2)	1.4 (1.3–1.5)	0.6

*Heart rate*			
Before IV access	87 (67–96)	80 (74–100)	0.8
During IV access	91 (80–115)	81 (72–95)	*0.02*
After IV access	82 (65–104)	82 (67–91)	0.5
*Blood pressure*			
Before IV access	113/75 (107/67–135/80)	109/70 (85/55–137/80)	0.4
During IV access	114/77 (110/68–122/80)	110/70 (98/60–120/80)	0.05
After IV access	110/75 (106/68–113/75)	103/72 (87/60–115/75)	0.3
*Saturation*			
Before IV access	98 (97–100)	97 (97–99)	0.9
During IV access	98 (98–100)	98 (97–100)	1.0
After IV access	98 (97–100)	98 (97–100)	1.0

**(b):** 

	EMLA + N2O	*p* ^*∗∗*^
*Control children*	(*n* = 30)	
*Age*, year	12 (5–18)	
*Gender*, male/female	21/9	
*BMI SDS*	5.0 (−1.2–8.9)	

*Pain*, 0–10	2.0 (1–10)	0.9
*17-OHP* nmol/L	2.7 (0.7–7.1)	<0.001
*Glucose* mmol/L	4.7 (4.1–5.2)	<0.01
*Norepinephrine* nmol/L	1.5 (0.9–2.7)	0.8

Data are presented as median and range. In the evaluation score, 5 is most satisfactory. ^*∗*^Wilcoxon signed rank test. ^*∗∗*^Comparing control children with CAH children treated with EMLA + N2O.
